# Neonatal Varicella-Zoster Virus Meningitis Without Pleocytosis: Diagnostic Value of Polymerase Chain Reaction

**DOI:** 10.7759/cureus.106504

**Published:** 2026-04-06

**Authors:** Abdelhakim Boulfouyoul, Kaoutar Ettoini, Abdallah Oulmaati

**Affiliations:** 1 Department of Pediatrics and Neonatology, Mohammed VI University Hospital Center, Tangier, MAR

**Keywords:** case report, cerebrospinal fluid, meningitis, neonatal varicella, pleocytosis, polymerase chain reaction, varicella-zoster virus

## Abstract

Neonatal varicella is uncommon but may lead to severe complications, including central nervous system involvement. Cerebrospinal fluid findings may be misleading in neonates, and polymerase chain reaction-confirmed viral central nervous system infection may occur without pleocytosis, particularly when lumbar puncture is performed early in the course of illness. We report a 17-day-old term male neonate admitted with generalized vesicular rash and fever associated with irritability and poor feeding. Lumbar puncture performed on admission showed normal cerebrospinal fluid parameters without pleocytosis; however, a multiplex cerebrospinal fluid polymerase chain reaction panel was positive for varicella-zoster virus, and vesicular lesion polymerase chain reaction confirmed varicella-zoster virus infection. The neonate received intravenous acyclovir for 21 days with complete clinical recovery and normal follow-up. This case highlights that a normal cerebrospinal fluid cell count does not exclude neonatal viral central nervous system infection and supports early cerebrospinal fluid polymerase chain reaction testing to confirm varicella-zoster virus central nervous system involvement while rapidly excluding major differentials such as neonatal herpes simplex virus infection.

## Introduction

Varicella-zoster virus infection in the neonatal period is uncommon but clinically important because disease severity depends on the timing of exposure and the degree of passive maternal antibody transfer [1,2]. Severe neonatal disease is most strongly associated with maternal varicella occurring close to delivery. The highest-risk window is typically described when maternal rash occurs from five days before delivery to two days after delivery [1,2]. In such situations, post-exposure prophylaxis with varicella-zoster immunoglobulin (VariZIG) is recommended for selected neonates at the highest risk, including certain premature infants after significant exposure [3]. Neonatal varicella can also be acquired postnatally through close household exposure, and complicated forms may occur outside the classic peripartum high-risk period.

Diagnosing neonatal central nervous system infection remains challenging because cerebrospinal fluid parameters may be noninformative early in the disease course. Although meningitis without cerebrospinal fluid pleocytosis is uncommon, it has been documented and may lead to false reassurance if clinicians rely primarily on cerebrospinal fluid leukocyte counts to exclude central nervous system infection [4]. In polymerase chain reaction-confirmed viral meningitis, particularly enteroviral meningitis, the absence of pleocytosis is well described, especially in young infants and when lumbar puncture is performed early [5,6]. Molecular diagnostic tools have therefore become essential in febrile neonates with neurological or systemic features, both to identify the causative pathogen and to rapidly exclude life-threatening differentials such as neonatal herpes simplex virus infection [7,8].

We report a neonate with postnatally acquired varicella complicated by varicella-zoster virus meningitis, confirmed by cerebrospinal fluid multiplex polymerase chain reaction despite a normal cerebrospinal fluid leukocyte count, highlighting the diagnostic value of early cerebrospinal fluid polymerase chain reaction testing in neonatal varicella.

## Case presentation

Patient information

A 17-day-old male neonate, born at term at 39 weeks' gestation, was admitted for fever associated with a generalized vesicular rash, irritability, and poor feeding. He was delivered by cesarean section for cephalopelvic disproportion after an uncomplicated pregnancy. Birth weight was 3380 g. Maternal history of prior varicella infection or varicella vaccination was not documented, and maternal immune status was unknown.

Household exposure was documented. The maternal uncle developed a varicella rash approximately one week before delivery and remained in close contact with the mother; close contact with the neonate continued after birth. The mother developed a varicella rash on day of life 11. Breastfeeding was ongoing, and the mother had vesicular nipple lesions at presentation.

Clinical findings

The illness began on day of life 14 with the appearance of a progressive rash. Three days later, on day of life 17, the neonate developed fever associated with irritability and poor feeding, prompting hospitalization.

**Figure 1:**
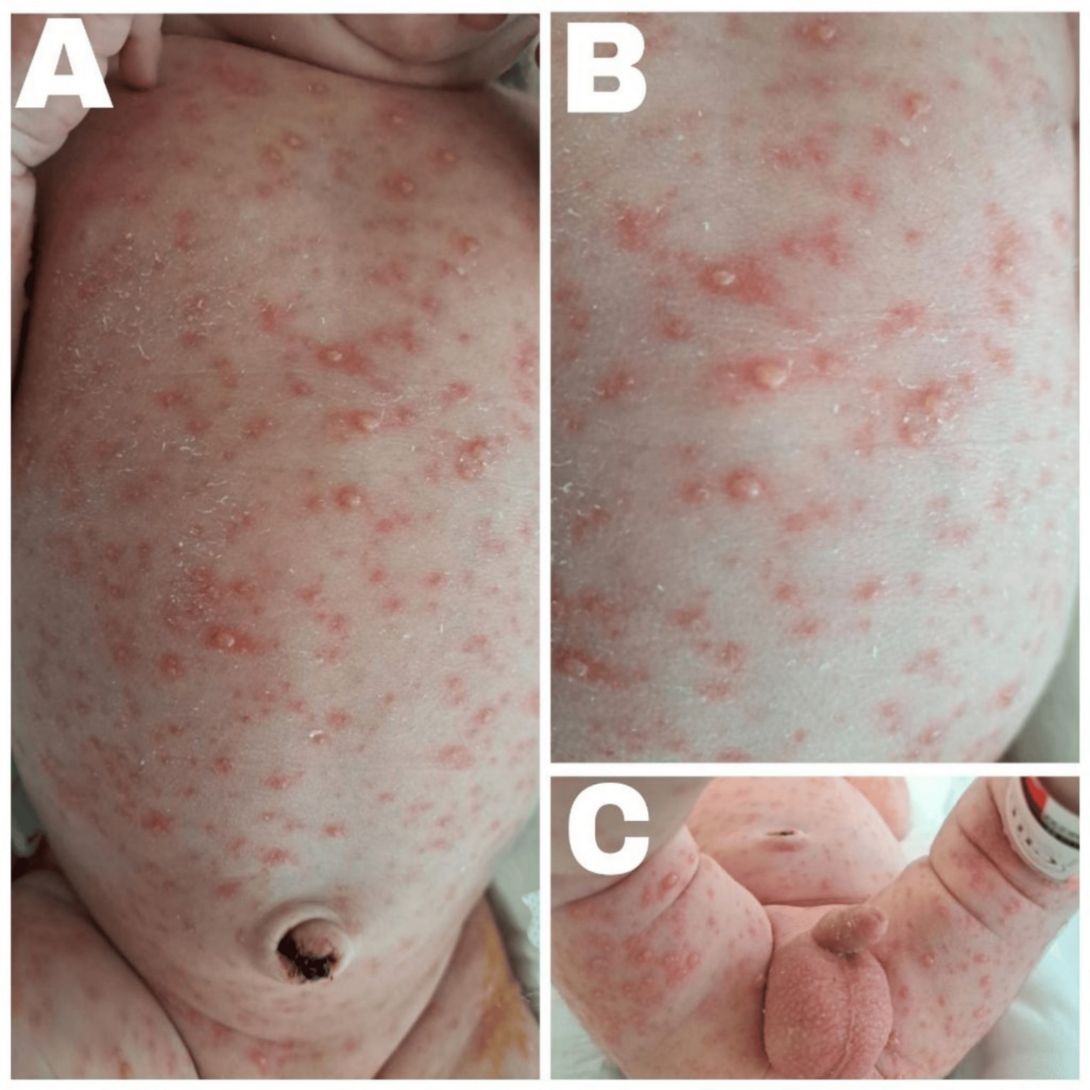
Cutaneous findings at presentation (A) Generalized erythematous papulovesicular eruption involving the trunk, with lesions in different stages of evolution. (B) Close-up view of the trunk showing multiple small vesicles on an erythematous base, consistent with active varicella lesions. (C) Involvement of the lower extremities and diaper area with scattered papulovesicular lesions, without mucosal involvement.

On admission, the neonate was febrile at 38.7°C and awake but irritable, with hypotonia and weak primitive reflexes. His weight was 4180 g, length was 52 cm, and head circumference was 37 cm, all appropriate for his age. Heart rate was 167 beats/min, respiratory rate was 47 breaths/min, and oxygen saturation was 97% on room air. There was no respiratory distress, pulmonary auscultation was normal, and the anterior fontanelle was soft and flat. Cutaneous examination revealed generalized papulovesicular lesions in different stages of evolution involving the face, scalp, trunk, and limbs, with sparing of the palms and soles and no mucosal involvement (Figure 1). Hemodynamic status was stable. No seizures, apnea, focal neurological deficits, or altered level of consciousness was observed.

Diagnostic assessment

A full sepsis and meningitis workup was performed, including evaluation for bacterial infection and neonatal herpes simplex virus infection.

Laboratory testing showed mild leukopenia, with a total leukocyte count of 4.5×10^3^/µL, an absolute lymphocyte count of 2.33×10^3^/µL, and an absolute neutrophil count of 1.81×10^3^/µL. Hemoglobin was 15 g/dL, with a C-reactive protein level of 1.2 mg/L. Platelet count, coagulation parameters, liver enzymes, electrolytes, and renal function were within normal limits. Urinalysis was unremarkable, and blood and urine cultures remained sterile. Chest radiography was normal.

Lumbar puncture performed on admission revealed clear cerebrospinal fluid with 2 white blood cells/µL and 1 red blood cell/µL, glucose of 45 mg/dL with concomitant blood glucose of 90 mg/dL, corresponding to a cerebrospinal fluid-to-serum glucose ratio of 0.5, and protein of 25 mg/dL. Cerebrospinal fluid culture remained negative.

A multiplex meningoencephalitis polymerase chain reaction panel performed on cerebrospinal fluid was positive for varicella-zoster virus and negative for other viral and bacterial targets, including herpes simplex virus. In addition, the varicella-zoster virus polymerase chain reaction performed on vesicular lesion fluid was positive. Cranial ultrasonography during hospitalization was normal.

Therapeutic intervention

Intravenous acyclovir was initiated after lumbar puncture at 20 mg/kg every eight hours and continued for 21 days.

Empiric intravenous antibiotics were also started on admission according to the local neonatal sepsis protocol, consisting of amoxicillin 100 mg/kg every 12 hours and gentamicin 3 mg/kg once daily. These were discontinued after 48 hours once bacterial infection became unlikely on the basis of sterile cultures and low inflammatory markers.

Supportive care included intravenous fluids with 10% dextrose because of poor feeding, paracetamol at 15 mg/kg every six hours as needed, and local skin care. Because the mother had vesicular nipple lesions, direct breastfeeding was temporarily withheld to avoid contact with active breast lesions, and the neonate was fed expressed breast milk by bottle as an alternative feeding method. Airborne and contact isolation precautions were implemented and maintained until the lesions crusted.

Follow-up and outcomes

The clinical course was favorable. Fever resolved within the first days of hospitalization. Irritability improved, tone normalized, and feeding progressively recovered by day 3 of antiviral treatment. No seizures, apnea, altered level of consciousness, or new neurological signs developed.

**Figure 2:**
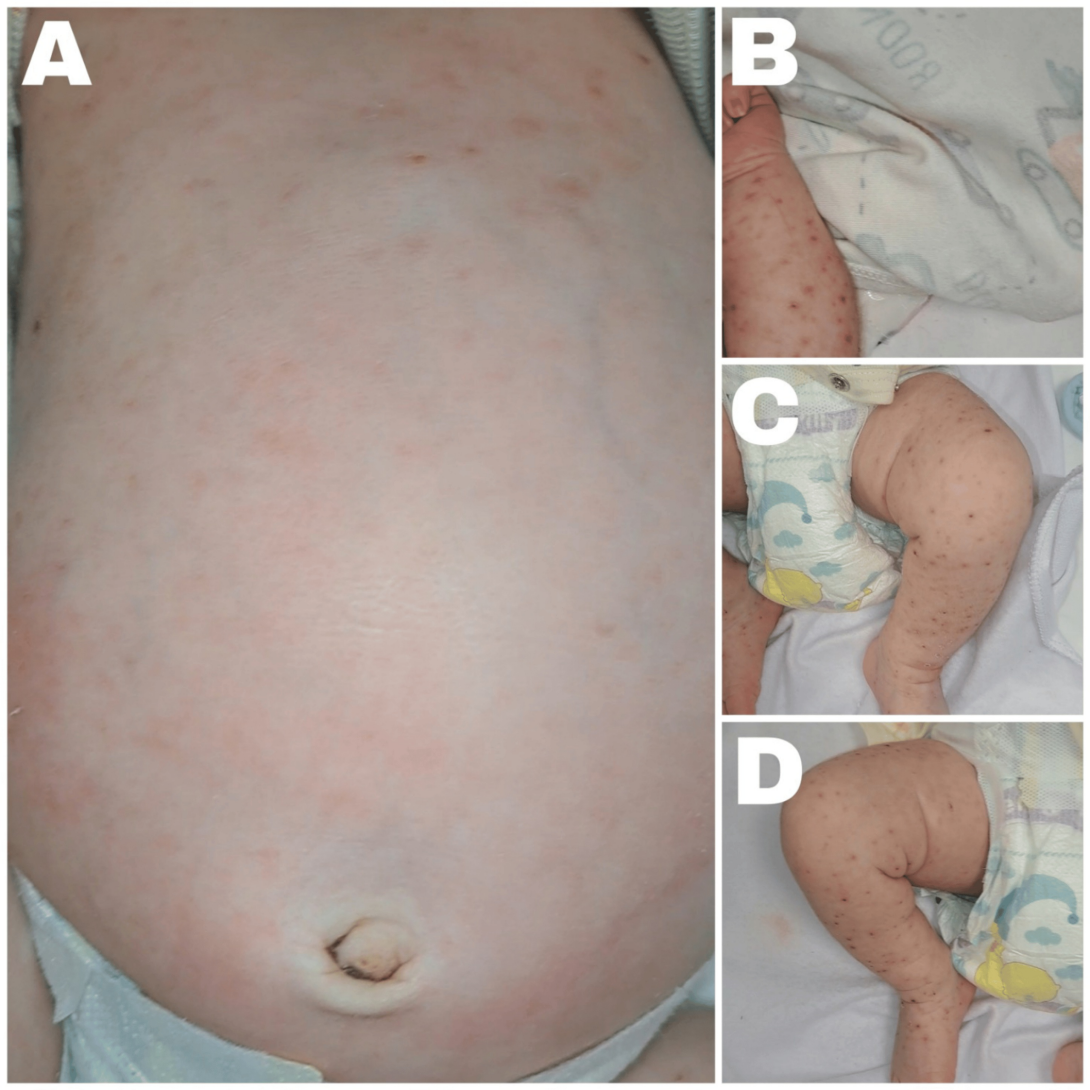
Evolution of cutaneous lesions under antiviral treatment on hospital day 9 (A) Trunk showing marked regression of the eruption, with the resolution of vesicles and persistence of faint erythematous macules. (B) Left upper limb showing scattered residual erythematous papules and macules, with no visible active vesicles. (C) Left lower limb demonstrating significant reduction in lesion density, with scattered residual erythematous papules. (D) Right lower limb illustrating the near-complete resolution of lesions, with residual erythema and the absence of active vesicular lesions.

The skin lesions progressively dried, and no new eruptions appeared, with marked regression by hospital day 9 and complete resolution by day 19 of antiviral therapy (Figure 2).

Laboratory monitoring remained reassuring throughout treatment, with stable platelet counts, liver enzymes, coagulation profile, and renal function. Electroencephalography, ophthalmologic examination, and hearing assessment were normal. Repeat lumbar puncture was not performed because of rapid clinical improvement without neurological deterioration, in line with the usual practice in our institution for similar clinically improving cases. The neonate completed 21 days of intravenous acyclovir and was discharged with outpatient follow-up.

Follow-up at one month, three months, six months, and 12 months showed normal growth and neurodevelopment without relapse or sequelae. Auditory brainstem response testing performed at six months was normal.

The clinical timeline is shown in Table 1.

**Table 1:** Clinical timeline of the case

Time point	Event
Approximate day 7	Maternal uncle developed a varicella rash and remained in close household contact with the mother
Day 0	Birth at term at 39 weeks' gestation
After birth	Close contact with the neonate continued
Day of life 11	Mother developed a varicella rash with vesicular nipple lesions
Day of life 14	Neonate developed a progressive rash
Day of life 17	Fever with irritability and poor feeding; admission; sepsis and meningitis workup; lumbar puncture; acyclovir and empiric antibiotics started
Hospital days 1-3	Defervescence and clinical improvement, with improved tone and feeding
Hospital day 9	Marked drying and regression of lesions (Figure 2)
Day 19 of antiviral therapy	Complete resolution and crusting of cutaneous lesions
End of therapy	Completed 21 days of intravenous acyclovir and was discharged
Follow-up	At one, three, six, and 12 months, normal growth and neurodevelopment; auditory brainstem response normal at six months

## Discussion

Neonatal varicella is rare but potentially severe, and its severity depends strongly on the timing of exposure relative to delivery and the passive transfer of protective maternal immunoglobulin G [1,2]. The period of highest risk for severe neonatal disease is classically defined when maternal rash develops from five days before delivery to two days after delivery [1,2], and post-exposure prophylaxis with VariZIG is recommended for selected neonates in that setting [3]. In the present case, the clinical context was outside this classic peripartum high-risk window.

The most likely transmission route was postnatal household exposure from the maternal uncle, given the documented close contact around the peripartum period and the continued contact after birth. Maternal rash developed later, on day of life 11, which may also have contributed to exposure, but the maternal uncle remains the most likely source.

One important diagnostic message of this case is that a normal cerebrospinal fluid analysis does not exclude neonatal central nervous system infection, particularly when lumbar puncture is performed early. Although meningitis without cerebrospinal fluid pleocytosis is uncommon, it has been documented and may lead to the premature exclusion of central nervous system infection if cerebrospinal fluid leukocyte counts are used as the sole decision criterion [4]. In polymerase chain reaction-confirmed viral meningitis, especially enteroviral meningitis, the absence of pleocytosis is well described and appears more frequent in early presentations and in younger infants [5,6]. While most published evidence concerns enterovirus, varicella-zoster virus meningitis with normal cerebrospinal fluid cellularity has also been reported [9]. In this case, early lumbar puncture may explain the absence of pleocytosis despite cerebrospinal fluid varicella-zoster virus polymerase chain reaction positivity, and the neurobehavioral symptoms supported clinically relevant central nervous system involvement [4-6,9].

Febrile neonates with a vesicular rash require the urgent exclusion of life-threatening conditions, particularly bacterial sepsis, bacterial meningitis, and neonatal herpes simplex virus infection [7]. Empiric antibiotics and intravenous acyclovir are commonly initiated while microbiological results are pending [7]. In this context, a multiplex cerebrospinal fluid polymerase chain reaction panel rapidly identified varicella-zoster virus while excluding herpes simplex virus and other major central nervous system pathogens, allowing the early refinement of management [8]. VariZIG was not administered because maternal varicella occurred well outside the classic highest-risk window of five days before to two days after delivery, and prophylaxis would not be routinely indicated for a term neonate in this setting [1,3]. Published data support the diagnostic utility of multiplex polymerase chain reaction assays for identifying central nervous system pathogens and guiding early management decisions [8]. In practice, polymerase chain reaction results should be interpreted in correlation with the clinical presentation; in this case, concordant vesicular lesion polymerase chain reaction positivity supported true varicella-zoster virus infection and reinforced the clinical relevance of the cerebrospinal fluid polymerase chain reaction result.

Neonates with suspected severe varicella or central nervous system involvement are generally treated with intravenous acyclovir [10]. In this case, acyclovir was initiated at the dose commonly used for neonatal herpes simplex virus central nervous system disease and continued for 21 days. Although treatment duration for neonatal varicella-zoster virus meningitis is less well standardized, a prolonged course was chosen because of cerebrospinal fluid polymerase chain reaction-confirmed central nervous system infection and associated neurobehavioral symptoms [10]. Supportive care, including hydration, feeding support, antipyretics, skin care, isolation precautions, and close monitoring, remains essential [10].

The favorable outcome in this case may be related to postnatal acquisition outside the highest-risk peripartum window, the absence of disseminated disease, and the prompt initiation of intravenous acyclovir [1,10].

## Conclusions

Neonatal varicella-zoster virus meningitis may occur without pleocytosis, particularly when lumbar puncture is performed early. In neonates with vesicular rash and systemic or neurobehavioral signs, cerebrospinal fluid polymerase chain reaction testing is valuable for establishing the diagnosis, excluding important differential diagnoses such as neonatal herpes simplex virus infection, guiding timely antiviral treatment, and avoiding the missed recognition of a potentially severe central nervous system infection.
